# Cardiac deceleration capacity is associated with severity of inflammation in COVID-19

**DOI:** 10.1007/s15010-023-02129-1

**Published:** 2023-11-27

**Authors:** John Michael Hoppe, Anna Katharina Strüven, Stefan Brunner, Christopher Stremmel

**Affiliations:** 1 Department of Medicine IV, LMU University Hospital, LMU Munich, Munich, Germany; 2 Department of Medicine I, LMU University Hospital, LMU Munich, Munich, Germany

**Keywords:** Deceleration capacity, Heart rate variability, Cardiac autonomic activity, Inflammation, SARS-CoV-2, COVID-19

## Abstract

**Purpose:**

In this pilot study, we investigated the cardiac autonomic activity of coronavirus disease 2019 (COVID-19)-infected hospitalized patients. COVID-19 is characterized by cough, fever, and dyspnea, which in some severe cases can lead to hypoxia, respiratory failure, and shock. Since breathing disorders and pulmonary diseases are tightly linked to autonomic dysfunction, we analyzed the cardiac autonomic activity by measuring the deceleration capacity (DC) in COVID-19 patients.

**Methods:**

In 14 adults (4 men and 10 women) with a median age of 63.5 years and positive for SARS-CoV-2 by polymerase chain reaction (PCR) with severe symptoms requiring hospital treatment, a high-resolution digital 30 min electrocardiogram (ECG) in Frank leads configuration was performed in a resting supine position within the first 48 h after hospital admission. DC was assessed using validated software and associated with several markers of inflammation and clinical course.

**Results:**

The study revealed a significant association between reduced DC (≤ 2.5 ms) and older age (74 years) in COVID-19 patients, compared to those with a higher DC > 2.5 ms (56.5 years). However, the duration of hospitalization was similar for both groups. There was a nonsignificant trend towards a higher maximum viral load in patients with reduced DC. Further, patients with a DC ≤ 2.5 ms showed higher levels of inflammatory markers such as C-reactive protein (CRP) and procalcitonin (PCT), as well as leukocytosis, compared to patients with a DC > 2.5 ms. Also, the COVID-19-severity marker ferritin was significantly elevated in patients with lower DC. Other markers associated with COVID-19, such as lactate dehydrogenase (LDH) and creatine kinase (CK), exhibited comparable levels in both groups.

**Conclusions:**

Reduced DC (≤ 2.5 ms) was significantly associated with older age, increased inflammatory markers, and elevated ferritin in patients with COVID-19. These findings suggest that DC might serve as a valuable indicator for predicting the risk of severe inflammation in COVID-19 and possibly complications associated with this disease, such as heart failure. Further studies are needed to confirm these observations and clarify the clinical significance of DC in COVID-19 and other infectious diseases.

## Introduction

Severe acute respiratory syndrome coronavirus type 2 (SARS-CoV-2) is a strain of Betacoronavirus that was first reported in late 2019 as the cause of multiple severe respiratory illness outbreaks in Wuhan City, China [1]. By January 2020, SARS-CoV-2 had been isolated and sequenced [2, 3], and after spreading worldwide and causing coronavirus disease 2019 (COVID-19), leading to numerous fatalities, the World Health Organization (WHO) declared on March 11, 2020, that COVID-19 should be characterized as a global pandemic [4]. Several studies have shown that COVID-19 can cause various heart conditions, such as myocarditis, pericarditis, cardiac arrhythmias, and cardiovascular diseases, among others [5–7]. Previous research has revealed that a number of heart rate variability (HRV) parameters, serving as surrogate markers for vagus nerve activity, predict mortality and severe illness in COVID-19 patients [8].

HRV analysis is a non-invasive and objective method that reflects the balance between the sympathetic and vagal branches of the autonomic nervous system. Deceleration capacity (DC) is a parameter of cardiac autonomic function that outmatches standardized methods of analyzing HRV concerning prognostic estimation [9]. Its calculation is based on transforming the series of beat-to-beat intervals (RR intervals) into a new time interval, the so-called phase rectified signal averaging (PRSA), which has been described in former publications [10]. PRSA has the advantage that the oscillations involved in the decelerations and accelerations of the heart can be observed separately from each other [11]. Hence, DC is an integral measure of all periodic oscillations influencing the deceleration of heart frequency. In turn, reduced DC implies impaired cardiac vagal modulations. DC has already been proven to be a powerful predictor of mortality in several diseases like myocardial infarction, heart failure, cancer, stroke, and pneumonia [9, 12–14]. Generally, DC lasting for less than 2.5 ms (ms) was defined as abnormal [15, 16].

Prior research has shown that compromised DC is associated with an increased risk of developing ARDS in persons with COVID-19 infection [14]. Nonetheless, since impaired cardiac autonomic function has been shown to be associated with inflammatory processes in the human body [17, 18], the significance of DC in predicting inflammation and disease severity in COVID-19 patients has yet to be explored.

The rationale of this pilot study was to investigate the impact of COVID-19 disease on cardiac autonomic function, namely the DC, as a marker of disease severity.

## Methods

### Study criteria and assessment of ECG-based measures of the cardiac autonomic system

In this pilot study, we sought to assess electrocardiogram (ECG)-based measures of the cardiac autonomic system in adults diagnosed with severe COVID-19. We included adults aged 18 years or older who tested positive for SARS-CoV-2 by PCR and required hospitalization. Our study population comprised 14 patients (4 men and 10 women) with a median age of 63.5 years. In all individuals, a high-resolution digital 30-min-ECG (Schiller Medilog AR4 plus, 1,000 Hz) was recorded in orthogonal Frank leads configuration in resting supine position under standardized conditions within the first 48 h from hospital admission. The ECG signals were analyzed using MATLAB with established algorithms for the calculation of DC, as previously published [9]. DC was chosen as a marker of unfavorable outcomes in individuals with COVID-19 since it is already known to be a robust predictor for morbidity and mortality in several infectious diseases, including hepatitis C, systemic sclerosis, and chronic obstructive pulmonary disease [19–21]. A DC of ≤ 2.5 ms has been described as a strong risk factor for cardiac mortality [9]. Therefore, DC dichotomization was performed at this cut-off value for group comparison.

### Statistical analysis

Statistical analysis was performed using Prism 9 software (GraphPad, USA). Continuous variables were reported as median and interquartile range (IQR). Mann–Whitney *U* test was used for group comparisons of nonparametric data. All tests were two-tailed and paired. *P* values < 0.05 were considered statistically significant. In this pilot study, we included all patients matching the inclusion criteria. No prior sample size calculation was performed.

## Results

### Baseline characteristics

In this pilot study, we enrolled 14 patients admitted to our COVID-19 ward between April 30th and May 26th, 2021. The median age was 63.5 years, and 10 of our patients were female. On average, symptoms persisted for 5.5 days before admission; the duration of hospitalization was 10 days; and patients presented with an average maximum viral load of 3.3 million copies per ml. The deceleration capacity was reduced on average to 2.75 ms, which has been described as a risk for heart failure [9]. Eight patients (57.1%) had a past medical history of hypertension, seven (50%) of pulmonary disease, seven (50%) of diabetes mellitus, and four (28.6%) of hypercholesterolemia (Table 1).

**Table 1:** Demographics and baseline characteristics

*n* = 14	
*Clinical parameters*	
Age (years)	63.5 (51.3; 73.8)
Male sex	4 (28.6%)
Female sex	10 (71,4%)
Symptoms before admission (days)	5.5 (3.3; 7.5)
Duration of hospitalization (days)	10 (9; 11)
ICU treatment	5 (35.7%)
Maximum oxygen demand—non ICU (l/min)	2 (2; 3)
Maximum viral load (copies/ml)	3.3 ∗ 10^6 (0.5 ∗ 10^6; 43 ∗ 10^6)
Deceleration capacity (ms)	2.75 (1.68; 3.51)
Deceleration capacity ≤ 2.5 ms	6 (42.9%)
Deceleration capacity > 2.5 ms	8 (57.1%)
*Medical history*	
Hypertension	8 (57.1%)
Pulmonary disease	7 (50%)
Diabetes mellitus	7 (50%)
Hypercholesterolemia	4 (28.6%)
Autoimmune disease	3 (21.4%)
Chronic kidney disease	3 (21.4%)
Hypothyroidism	2 (14.3%)
Atrial fibrillation	2 (14.3%)
*Laboratory findings (maximum values)*	
CRP (mg/dl)	7.5 (5.7; 16.1)
IL-6 (pg/ml)	62.1 (24.1; 99.7)
PCT (ng/ml)	0.1 (0.1; 0.4)
Leukocytes (G/l)	11.3 (8.4; 14.9)
Ferritin (ng/ml)	545 (310; 995)
LDH (U/l)	421 (332; 571)
CK (U/l)	148 (79; 193)

Data are median (IQR) or *n* (%).

*ICU* intensive care unit; *CRP* C-reactive protein; *IL-6* interleukin 6; *PCT* procalcitonin; *LDH* lactate dehydrogenase; *CK* creatine kinase

All patients received 6 mg of dexamethasone for 10 days (or until discharge) and venous thromboembolism prophylaxis until discharge. Five patients received intensive care treatment, and one patient died from COVID-19. Patients that did not require intensive care had a median oxygen demand of 2 l/min. As expected, COVID-19 patients presented with elevated inflammatory markers such as C-reactive protein (CRP) and leukocytosis. Further, ferritin, lactate dehydrogenase (LDH), and creatine kinase (CK) were also elevated (Table 1).

### Reduced deceleration capacity is associated with older age in COVID-19

**Fig. 1:**
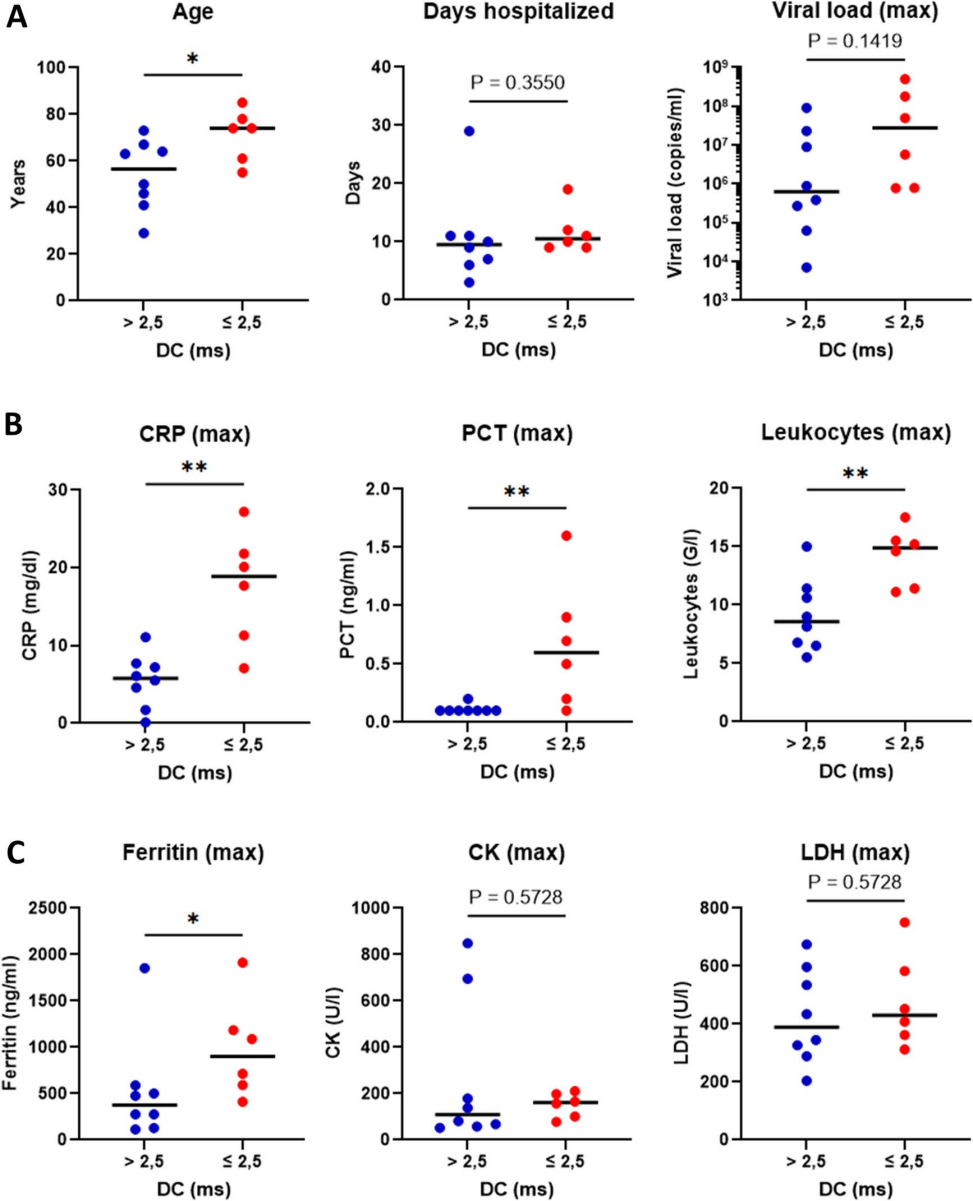
Increased inflammatory markers and adverse baseline characteristics displayed in patients with reduced deceleration capacity (DC) of ≤ 2.5 ms compared to > 2.5 ms. **A** Age in years, duration of hospitalization in days, and viral load in copies/ml. **B** Maximum values of inflammatory markers CRP in mg/dl, PCT in ng/ml, and leukocytes in G/l. **C** Maximum values of COVID-19-severity markers ferritin in ng/ml, CK in U/l and LDH in U/l. Data are expressed as median. *n* = 6 to 8 per group. * *p*< 0.05, ** *p* < 0.01, exact *p* values are shown for non-significant data. *CRP* C-reactive protein; *IL-6* interleukin 6; *PCT* procalcitonin; *LDH* lactate dehydrogenase; *CK* creatine kinase

COVID-19 patients with a high-risk DC of ≤ 2.5 ms (*n* = 6) were compared to those presenting a lower-risk DC of > 2.5 ms (*n* = 8). The analysis revealed that a DC ≤ 2.5 ms was significantly associated with an older median age of 74 years, whereas a DC > 2.5 ms was associated with a younger median age of 56.5 years (Fig. 1). Markedly, there was a tendency towards a higher maximum viral load in patients with reduced DC. 27 × 10^6^ copies/ml in patients presenting with a DC ≤ 2.5 ms compared to 0.6 × 10^6^ copies/ml in patients with a DC > 2.5 ms. However, this finding was not statistically significant. The duration of hospitalization was similar for both groups, 10.5 days for DC ≤ 2.5 ms and 9.5 days for DC > 2.5 ms (Fig. 1A).

### Reduced deceleration capacity is associated with elevated inflammatory markers and ferritin in COVID-19

Patients with a DC ≤ 2.5 ms exhibited significantly higher levels of inflammatory markers compared to patients with a DC > 2.5 ms. Notably, median C-reactive protein (CRP) levels were measured at 18.9 mg/dl in patients displaying a DC ≤ 2.5 ms, whereas patients with a DC > 2.5 ms presented with CRP levels of 5.8 mg/dl. Similarly, in patients with a DC of ≤ 2.5 ms, both procalcitonin (PCT) and leukocyte levels were significantly higher, at 0.4 ng/ml and 14.9 G/l, respectively. In contrast, those with a DC > 2.5 ms had on average PCT levels of 0.1 ng/ml and normal range leukocytes at 8.6 G/l (Fig. 1B). Furthermore, in patients presenting with a DC of ≤ 2.5 ms, ferritin, a marker associated with COVID-19 severity [22], was significantly elevated at 901 ng/ml compared to 376 ng/ml in those with a DC > 2.5 ms. However, lactate dehydrogenase (LDH) and creatine kinase (CK) were the same in both groups (Fig. 1C).

## Discussion

This pilot study enrolled 14 patients admitted to our COVID-19 ward, and we observed several key findings in regards to the baseline characteristics and clinical outcomes of these patients. Typical for COVID-19-associated hospitalization, the median age of our patients was 63.5 years. On average, the duration of symptoms before admission was 5.5 days, and the duration of hospitalization was 10 days. Patients presented with an average maximum viral load of 3.3 million copies per ml, and the deceleration capacity (DC) was reduced on average to 2.75 ms, previously described as a risk factor for mortality in patients after myocardial infarction and for heart failure [9, 23].

Uniquely, DC quantifies all deceleration-related periodic modulations of heart rate, regardless of their frequency and origin. Therefore, DC is a robust measure of autonomic nervous system integrity that strongly correlates with a patient's overall prognosis without being restricted to a specific disease mechanism [24]. In the present study, we found that older age was significantly associated with reduced DC in COVID-19, with patients aged 74 years having lower DC (≤ 2.5 ms) compared to patients aged 56.5 years (DC > 2.5 ms). Notably, advanced age (> 60 years) has been described as a prominent risk factor for mortality in COVID-19 [25, 26]. Although there was a tendency towards a higher maximum viral load in patients with reduced DC, this association did not reach statistical significance. The duration of hospitalization was similar between the two groups, indicating that DC may not be a determinant of hospitalization duration in COVID-19 patients. However, it should be taken into consideration that the only patient who died of COVID-19-related causes was part of the low DC group, skewing the average hospitalization towards a shorter duration for this group.

Importantly, our study revealed that patients with a DC of ≤ 2.5 ms exhibited higher levels of inflammatory markers compared to patients with a DC > 2.5 ms. In detail, levels of CRP and PCT were significantly elevated in patients with reduced DC, indicating a higher inflammatory burden. In addition, patients with a DC ≤ 2.5 ms presented with leukocytosis, further supporting the association between reduced DC and increased inflammatory response in COVID-19. Furthermore, ferritin, a widely recognized biomarker for predicting COVID-19 severity [22], displayed a significantly higher elevation in patients with a DC ≤ 2.5 ms. This indicates that DC reduction could also serve as a marker for COVID-19 severity. Moreover, these observations suggest a potential link between DC reduction and altered cardiac iron metabolism in COVID-19. This is supported by studies highlighting that hyperferritinemia, dysregulated iron metabolism, and cardiac iron overload are associated with severe COVID-19 courses [27].

Our findings are consistent with previous studies that have reported the association between reduced DC and older age as well as increased inflammatory markers in various clinical settings [28]. For example, in heart failure patients, reduced DC has been shown to be predictive of adverse outcomes and increased mortality [9]. Further, our study adds to the growing body of evidence suggesting that autonomic activity may also serve as a potential marker of inflammatory status in COVID-19 patients [18].

The absence of a notable elevation in LDH among patients with a DC ≤ 2.5 ms could be attributed to the relatively mild presentation of COVID-19 symptoms in our cohort. Only five of the 14 patients required intensive care treatment. Previous studies on COVID-19 have indicated that LDH levels tend to be significantly elevated in patients necessitating intensive care compared to those on normal wards and that LDH is an early predictor of severe disease progression [29, 30].

One limitation of this pilot study is its small sample size. Thus, larger prospective studies are necessary to validate our findings and elucidate the underlying mechanisms linking reduced DC with inflammatory response in COVID-19. Also, the relatively wide dispersion of the individual time between the onset of symptoms and the presentation to the hospital impedes the comparability of the measurement results. Further, more women were enrolled than men, which may limit the representativeness of the data for the male demographic.

Nonetheless, our study underlines the potential clinical significance of DC as a noninvasive marker of inflammatory status in COVID-19 patients, which may have implications for risk stratification and management strategies in this population. Therefore, future studies should explore the use of DC as a prognostic tool and its potential implications for patient care in the context of COVID-19 and other infectious diseases.
